# T112. TRADITIONAL RISK FACTORS NOT ENOUGH TO EXPLAIN THE SHORT LIFETIME EXPECTANCY IN PATIENTS WITH SCHIZOPHRENIA

**DOI:** 10.1093/schbul/sby016.388

**Published:** 2018-04-01

**Authors:** Moradi Hawar, Anna-Karin Olsson, Fredrik Hjärthag, Madeleine Johansson, Maivor Olsson-Tall, Lars Helldin

**Affiliations:** 1 Karlstad University; 2 NU Health Care Hospital, Karlstad University; 3 NU Health Care Hospital

## Abstract

**Background:**

Patients with schizophrenia have about 20 years shorter lifetime expectancy compared to healthy population. The cause of this excess in mortality is due to both unnatural and natural causes. While the lifetime prevalence of death due to suicide among patients with schizophrenia is estimated to be 4.9%, Cardiovascular (CV) disease contributes to as much as 50% of the excess mortality in patients with schizophrenia.

This study focuses on whether hypertension, diabetes, hyperlipidemia and tobacco could be related to the reduced lifetime expectancy in patients with schizophrenia spectrum disorder.

**Methods:**

From the Clinical Long-term Investigation of Psychosis in Sweden (CLIPS) study, 79 patients now deceased were analyzed at baseline. Data regarding occurrence of hypertension, diabetes, hyperlipidemia, tobacco but also data on the type of antipsychotic treatment were collected. Two patients, one with zero risk factors and one with 5 risk factors were omitted from the study. We created four categories based on the number of risk factors. 31 patients with one risk factor, 24 patients with two risk factors, 12 patients with three risk factors and 4 patients with four risk factors.

**Results:**

The mean age for death was 61 years and the age varied between 35–83 years old. 18 percent were treated with typical antipsychotics and 61 percent with atypical antipsychotics. 18 percent had both atypical and typical antipsychotic treatment. 17 percent had treatment for diabetes, 27 percent had treatment for hypertonia, 8 percent had treatment for hyperlipidemia and 43 percent were using tobacco. The data collected pictures the occurrence of the different risk factors on average 6 years before their death. We compared the age of death for the four different risk factor groups with a Kruskal-Wallis Test and could not find any significant difference between them.

**Discussion:**

Compared to the general population in Sweden there is an increased risk for diabetes in patients with schizophrenia, however the prevalence of hypertonia is the same, 27 percent for 18 years old and elder, in the general population. Daily tobacco use was rather high among patients with schizophrenia. Compared to general population, women and man with 10 percent respective 8 percent higher. Even if both diabetes and tobacco use has a high prevalence in patients with schizophrenia, it may not be enough to explain the reduced lifetime expectancy in patients with schizophrenia This study indicates that metabolic syndrome and the risk factors it contains need to be further studied in order to find its association to early death in patients with schizophrenia.

